# Ambient Air Pollution Exposure and Respiratory, Cardiovascular and Cerebrovascular Mortality in Cape Town, South Africa: 2001–2006

**DOI:** 10.3390/ijerph9113978

**Published:** 2012-11-02

**Authors:** Janine Wichmann, Kuku Voyi

**Affiliations:** 1 School of Health Systems and Public Health, Health Sciences Faculty, University of Pretoria, P.O. Box 667, Pretoria 0001, South Africa; Email: kuku.voyi@up.ac.za; 2 Section of Environmental Health, Health Sciences Faculty, University of Copenhagen, Øster Farimagsgade 5A, 1014 Copenhagen, Denmark

**Keywords:** air pollution, particulate matter, nitrogen dioxide, sulfur dioxide, respiratory, cardiovascular, cerebrovascular, mortality, case-crossover, South Africa

## Abstract

Little evidence is available on the strength of the association between ambient air pollution exposure and health effects in developing countries such as South Africa. The association between the 24-h average ambient PM_10_, SO_2_ and NO_2_ levels and daily respiratory (RD), cardiovascular (CVD) and cerebrovascular (CBD) mortality in Cape Town (2001–2006) was investigated with a case-crossover design. For models that included entire year data, an inter-quartile range (IQR) increase in PM_10_ (12 mg/m^3^) and NO_2_ (12 mg/m^3^) significantly increased CBD mortality by 4% and 8%, respectively. A significant increase of 3% in CVD mortality was observed per IQR increase in NO_2_ and SO_2_ (8 mg/m^3^). In the warm period, PM_10_ was significantly associated with RD and CVD mortality. NO_2_ had significant associations with CBD, RD and CVD mortality, whilst SO_2_ was associated with CVD mortality. None of the pollutants were associated with any of the three outcomes in the cold period. Susceptible groups depended on the cause-specific mortality and air pollutant. There is significant RD, CVD and CBD mortality risk associated with ambient air pollution exposure in South Africa, higher than reported in developed countries.

## 1. Introduction

Chronic obstructive airways disease (COPD), cardiovascular disease (CVD) and cerebrovascular disease (CBD) incidence is increasing in South Africa, as in many developing countries [1]. The short-term effects of particles with a 50% cut-off at an aerodynamic diameter of 2.5 μm or less (PM_2.5_), PM_10_, carbon monoxide (CO), nitrogen dioxide (NO_2_), ozone (O_3_), sulfur dioxide (SO_2_) on respiratory disease (RD), CVD and CBD mortality and morbidity were summarized in a meta-analysis [2]. The majority of the case-crossover or time series epidemiological studies included in the meta-analysis were conducted in North America and Western Europe, with more from Asia and South America since 2004. 

There remains a need for similar studies and long-term cohort studies in cities of developing countries, as levels and composition of air pollution are different from North America and Western Europe due to different sources [3]. Differences in the vulnerability of the population, building characteristics, time-activity patterns and proximity to air pollution sources may also modify the effects of exposure. A better understanding is also needed of how air pollution from indoor sources contributes to levels of outdoor air pollution and how indoor exposure to air pollution from indoor sources influences risk estimates for outdoor air pollution.

Exposure to outdoor air pollutants is essentially beyond the control of individuals and requires action by public authorities at the national, regional and even international levels. Outdoor air quality remains a key issue in Cape Town [4]. The main sources of PM_10_ are diesel vehicles, wood and coal burning for household heating and cooking fuel, dust from construction activities and unpaved roads, as well as wind-blown dust. The main sources of NO_2_ and SO_2_ are diesel vehicles and coal burning for household fuel, as there are few large industries in the city [4].

The objective of this study was to investigate if there is a significant association between the 24-h average ambient PM_10_, SO_2_ and NO_2_ levels and daily RD, CVD and CBD mortality in Cape Town, South Africa during 2001–2006.

## 2. Methods

### 2.1. Study Design

The association between daily PM_10_, SO_2_ and NO_2_ levels and daily RD, CVD and CBD mortality was investigated with the case-crossover epidemiological design [5]. The case-crossover design was developed as a variant of the case-control design to study the effects of transient exposures on emergency events, comparing each person’s exposure in a time period just prior to a case-defining event with person’s exposure at other times. Hereby, control on all measured and unmeasured personal characteristics that do not vary over a short time period is accomplished. If in addition, the control days are chosen close to the event day, personal characteristics that vary slowly over time are also controlled by matching. We applied a time-stratified approach to select the control days, defining the date of death as the case day and the same day of the week in the same month and year as control days [6,7].

### 2.2. Mortality Data

Data (all ages) for the period 1 January 2001–31 December 2006 in the Cape Town metropolitan municipality were obtained from the Health Information and Technology department, City of Cape Town (See Appendix). Groenewald *et al.* reported on the descriptive statistics of the mortality data [8]. This study focused on the following causes of death (International Classification of Diseases 10th Revision codes in parentheses): RD deaths: Pneumonia (J12–J18), bronchitis (J40–J42), COPD (J44), emphysema (J43), asthma (J45–J46), respiratory distress syndrome (J80), other diseases of the respiratory system (J95, J97–J99), respiratory failure (J96). CVD deaths: Acute rheumatic fever (I00–I02), chronic rheumatic heart disease (I05–I09), hypertensive disease (I10–I15), ischemic heart disease (I20–I25), pulmonary heart and circulatory diseases (I26–I28), other heart diseases (I30–I43, I51–I52), heart failure (I50). CBD deaths: All cerebrovascular diseases (I60–I69).

### 2.3. Air Pollution Data

**Figure A1:**
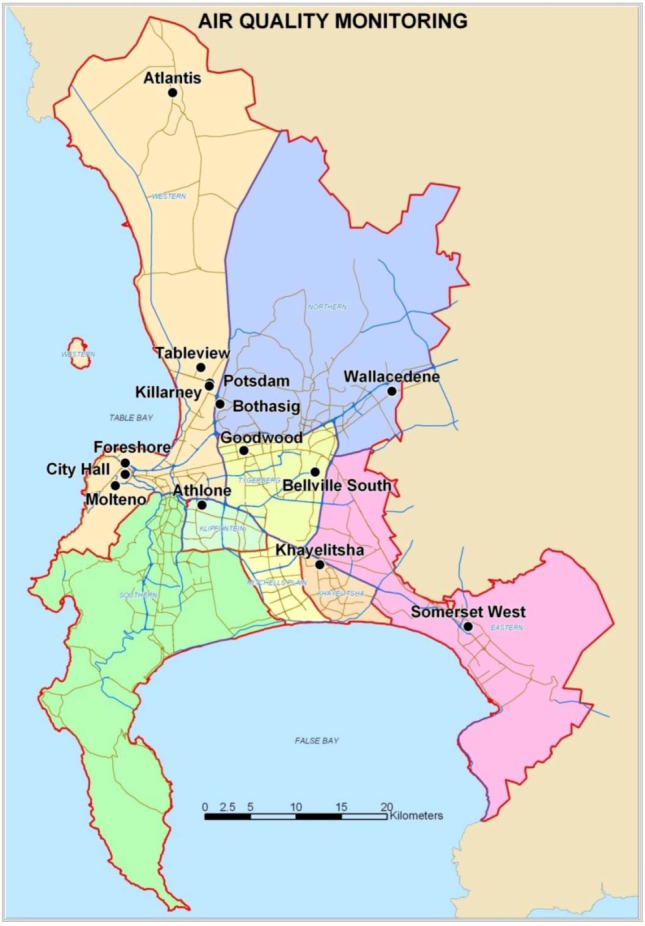
Map of the air quality monitoring network in Cape Town, South Africa during 2012 [4].

Data for the period 1 January 2001–31 December 2006 were provided by the Air Quality Monitoring Laboratory, City of Cape Town as 1-hour averages. A network of analyzers located at nine fixed sites and two mobile stations continuously assess real-time concentrations of the criteria air pollutants using equivalent methods of the United States Environmental Protection Agency and in accordance with ISO 17025 guidelines (See Appendix, Figure A1). The National Environmental Management: Air Quality Act of 2004 requires the monitoring of PM_10_, NO_2_ and SO_2_ [9]. These three air pollutants were included in the study and measured at the City Centre, Goodwood and Tableview sites. Data from the other sites were not used due to the large number of days with missing values.

Daily 24-hour averages for the three sites were calculated from the hourly data and are based on at least 20 1-hour values in accordance with the ISO 17025 guidelines. Subsequently an aggregated 24-h average for each of the three pollutants was calculated across the three sites. Such an approach was applied in the large Air Pollution and Health: A European Approach project [10].

### 2.4. Confounder Data

Influenza data were not available. The statistical analyses were therefore conducted for the entire year, the warm months (January–April, September–December) and cold months (May–August). 

Daily temperature and relative humidity data were obtained from the South African Weather Services for the period 1 January 2001–31 December 2006. Temperature (°C), relative humidity (%), wind speed (km·h^−1^) and rainfall (mm) data were recorded at Cape Town International Airport, which is located about 25 km from the city center. Meteorological data were not recorded at the air pollution monitoring sites.

According to Barnett *et al.* there is no single temperature measure that is superior to others [11]. In this study temperature and relative humidity were adjusted for as apparent temperature (Tapp), which is a construct intended to reflect the physiological experience of combined exposure to humidity and temperature and thereby better capture the response on health than temperature alone [12] (See Appendix). Apparent temperature has been applied in several studies [13,14,15].

### 2.5. Statistical Analysis

The case-crossover epidemiological design was applied to investigate the association between air pollution and cause-specific mortality. A time-stratified approach was applied to select the control days, defining the day of death as the case day and same day of the week in the same month and year as control days.

We analyzed the data using conditional logistic regression analysis (PROC PHREG in SAS 9.2, SAS Institute, Cary, NC, USA). Previous studies reported a linear relationship between the air pollutants and the cause-specific deaths [2]. PM_10_, SO_2_, NO_2_ were modeled as linear terms, one pollutant at a time. 

Evidence indicates that short-term air pollution exposures are most appropriate to detect health effects in time series and case-crossover studies [2]. There is no standard method to select lags [2]. Most studies select a lag that is significant and has the lowest Akaike Information Criterion (AIC). Lag0 (same day exposure as day of death) to lag5 (exposure five days prior to day of death) were investigated, as well as accumulated exposures: mean of lag0-1 (2-day simple unweighted cumulative average, CA2), and up to mean lag0-5 (CA6). Control days for lag1 to lag5 were defined as for lag0. 

Models were controlled for confounding by public holidays (binary variable) and the meteorological variables (same lag as a pollutant): Tapp, rainfall and wind speed. 

Odds ratios (OR) and the 95% confidence intervals (CI) were calculated per inter-quartile range (IQR) increase in the pollutant levels. The results are presented as the percent excess risk in cause-specific mortality per IQR increase in the pollutant levels using the following calculation: (exp^(IQR × β)^ – 1) × 100%, where β is the model estimate.

We stratified the models by sex, age groups (≤60 years and >60 years) or distance from the three monitoring sites to investigate susceptible groups. Suburbs where deaths occurred were stratified on distance from the three sites, in order to address possible exposure misclassification: ≤10 km (320 out of 485 suburbs) and >10 km (165 out of 485 suburbs). 

The linearity of the association between Tapp and a cause-specific outcome was confirmed in generalised additive Poisson time-series regression models (GAM) with the use of the *gam* procedure, *mgcv* package in R statistical software (R Development Core Team, 2010). Smoothing splines of calendar time with 2 to 8 degrees of freedom per year (df/year) were used to control for long-term trend and seasonality, with between 4 and 8 df/year the optimum for the entire year, and the warm and cold periods. Models were run with linear and non-linear terms of Tapp, the latter as a smoothing spline function with 3, 5 and 7 df. We investigated whether the non-linear term of Tapp improved the models by conducting log-likelihood ratio tests. We decided not to use a spline for Tapp, as the splines were insignificant, did not add value to the models and the pollutant model estimates were not influenced, whether 3, 5 or 7 df. The GAM models were also adjusted for day of the week (dummy variable), public holidays (binary variable), wind speed and rainfall (linear variables).

### 2.6. Ethics

This study was purely registry based; no human participants were recruited or included in experiments. 

## 3. Results

### 3.1. Mortality

A total of 149,667 total deaths (ICD10 A00–Z99) (all ages) occurred in the entire Cape Town metropolitan municipality during 2001–2006. The top five causes of death were unintentional and intentional injuries (ICD10 ≥ R99), CVD, infectious and parasitic diseases (including HIV/AIDS) (ICD10 A00–B99), neoplasms (cancers) (ICD10 C00–D48) and RD. In this study, 13,439 RD; 21,569 CVD and 7,594 CBD deaths were included in the analyses.

**Figure A2:**
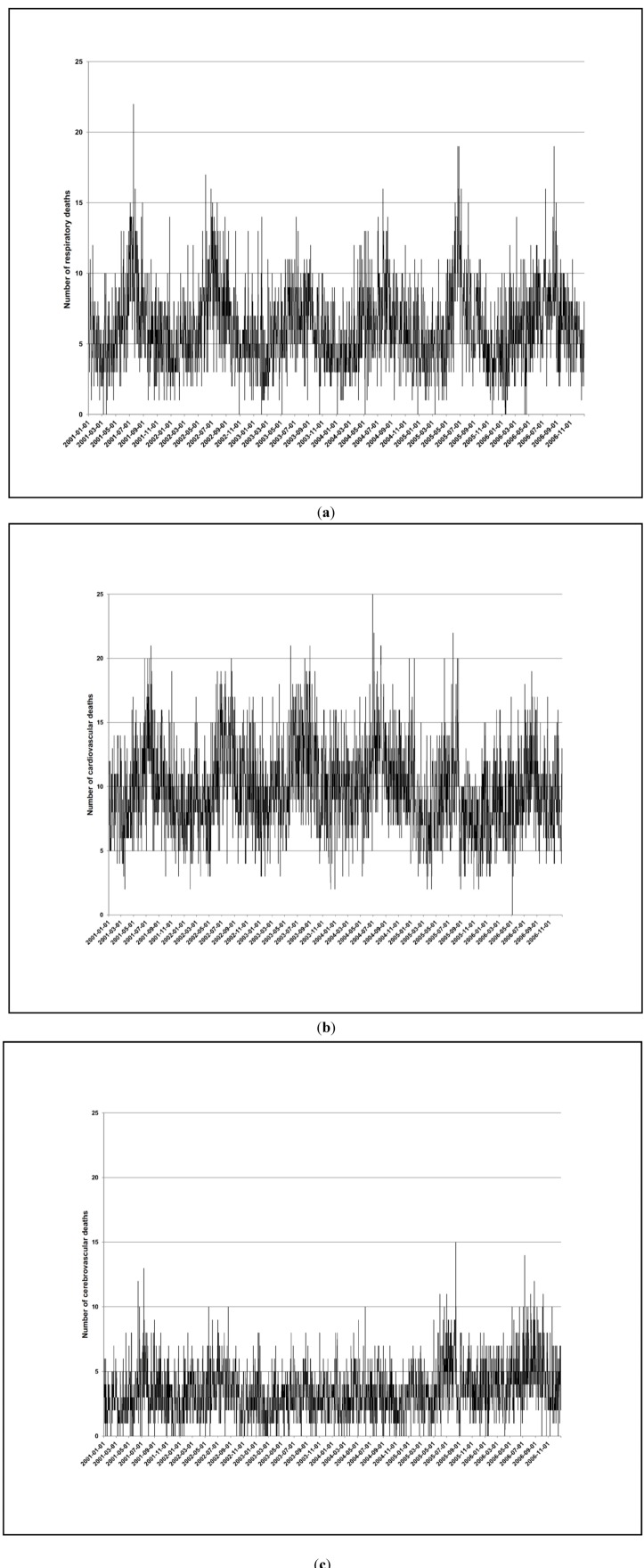
Time series of respiratory (**a**), cardiovascular (**b**) and cerebrovascular mortality (**c**) in Cape Town during 1 January 2001–31 December 2006.

Figure A2 (see Appendix) illustrates the time series of cause-specific mortality. The pattern of cause-specific mortality varied as expected by season, with a higher number of deaths on average per month during the cold period (24 months out of 72 months). Table 1 summarizes the characteristics of the cause-specific deaths. The mean age for RD mortality was 58 years, and for CVD and CBD mortality it was 69 years. For the ≤60 year group, the majority of RD deaths were due to pneumonia (39%), chronic obstructive pulmonary disease (COPD) (20%), other diseases of the respiratory (14%) and asthma (14%). For the >60 year group, the majority of RD deaths were due to pneumonia (36%), COPD (32%), other diseases of the respiratory (10%), asthma (8%) and emphysema (7%). Regardless of age group, the majority of CVD deaths were due to ischemic heart disease (55%), hypertensive disease (17%) and heart failure (18%).

**Table 1:** Characteristics of cause-specific mortality in Cape Town, South Africa (1 January 2000–31 December 2006).

	Respiratory Disease	Cardiovascular Disease	Cerebrovascular Disease
	*N* (%)	*N* (%)	*N* (%)
**Total**	13,439 (100)	21,569 (100)	7,594 (100)
**Period** **^a^**			
Warm	7,647 (57)	13,229 (61)	4,580 (60)
Cold	5,792 (43)	8,340 (39)	3,014 (40)
**Sex**			
Male	7,398 (55)	10,396 (48)	3,372 (44)
Female	6,041 (45)	11,173 (52)	4,222 (56)
**Age**			
≤60 years	6,146 (46)	6,003 (28)	2,100 (28)
>60 years	7,293 (54)	15,566 (72)	5,494 (72)
**Age and sex**			
≤60 years, male	3,630 (27)	3,555 (16)	1,091 (14)
≤60 years, female	2,516 (19)	2,448 (11)	1,009 (13)
>60 years, male	3,768 (28)	6,841 (32)	2,281 (30)
>60 years, female	3,525 (26)	8,725 (40)	3,213 (42)
**Distance from monitoring sites ^b^**			
≤10 km	6,986 (52)	12,931 (60)	4,583 (60)
>10 km	6,453 (48)	8,638 (40)	3,011 (40)

^a^ Warm period: January–April and September–December, cold period May–August; ^b^ City Centre, Goodwood and Tableview

### 3.2. Air Pollution

**Figure A3:**
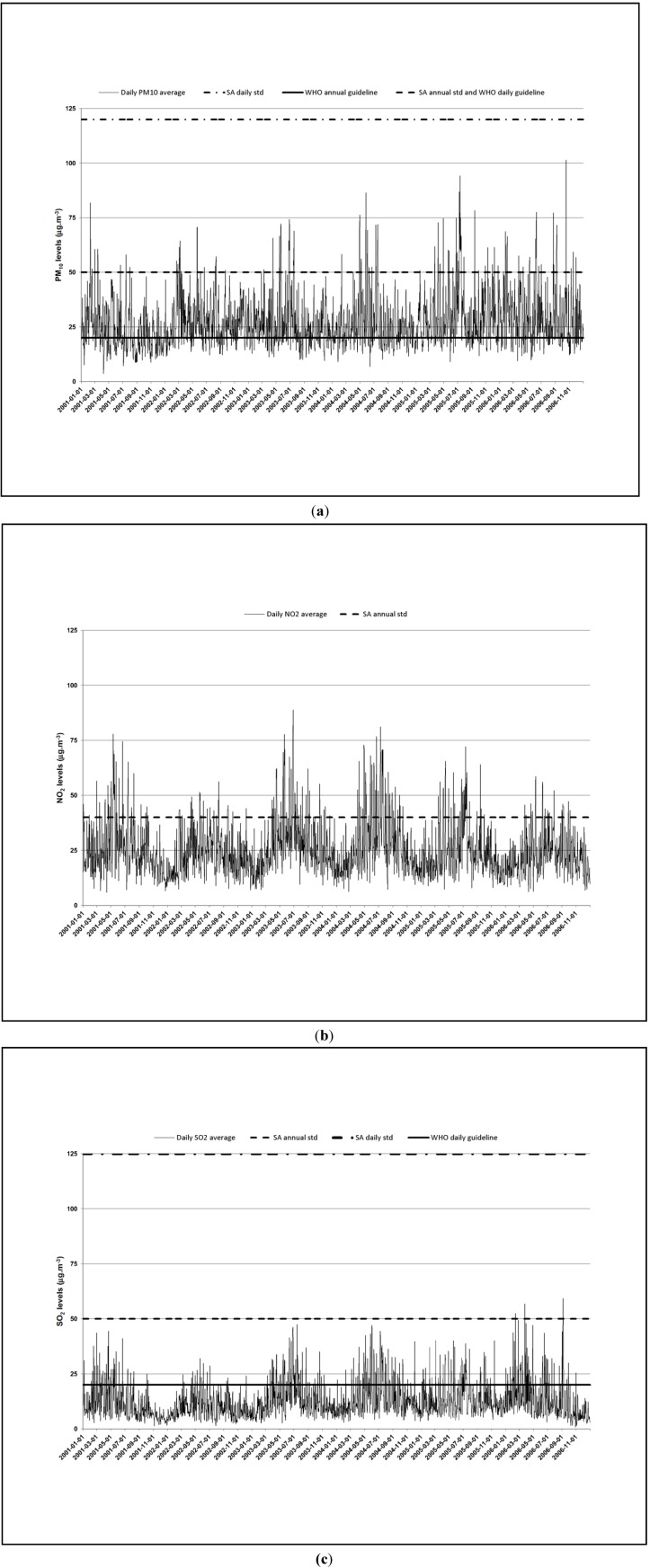
Time series of PM_10_ (**a**), NO_2_ (**b**) and SO_2_ (**c**) in Cape Town during 1 January 2001–31 December 2006.

**Figure A4:**
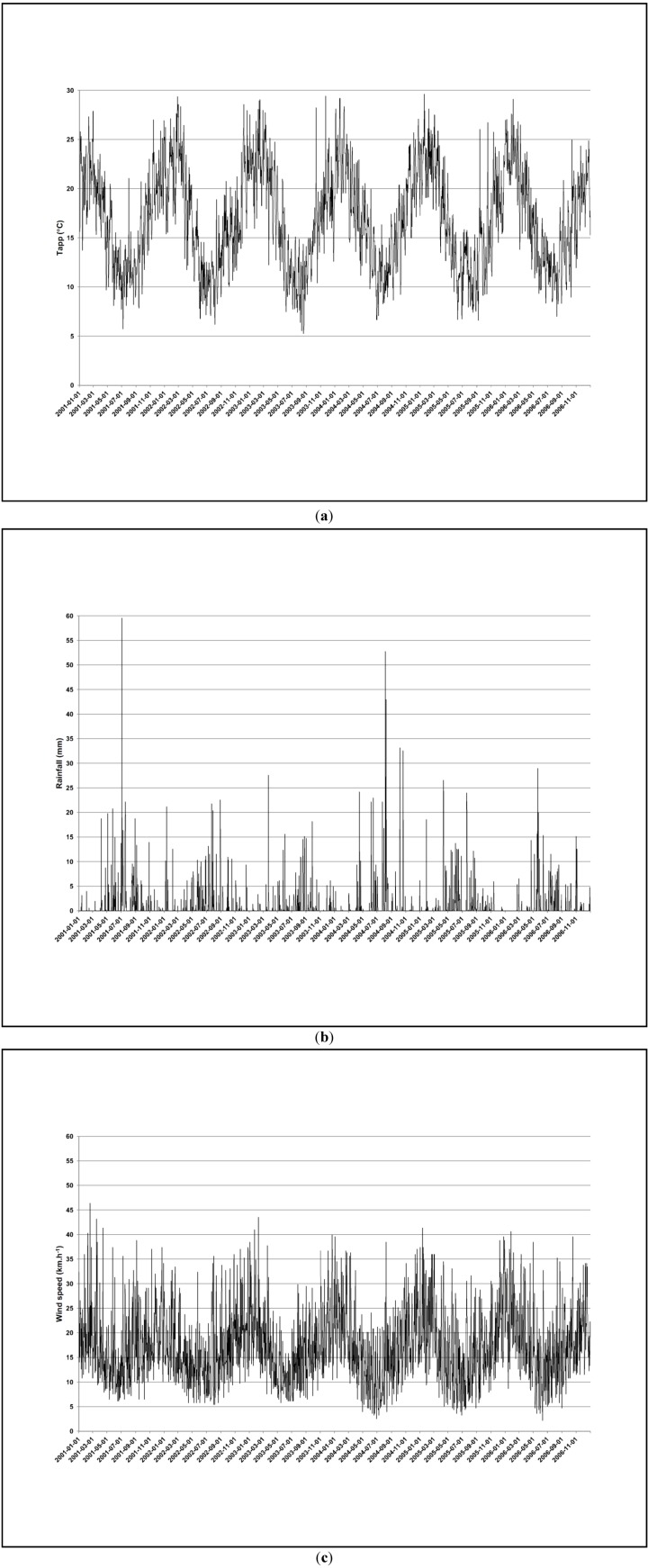
Time series of Tapp (**a**), rainfall (**b**) and wind speed (**c**) in Cape Town during 1 January 2001–31 December 2006.

Figure A3 and Figure A4 (see Appendix) illustrate the time series of the air pollutants and meteorological variables. NO_2_ and the meteorological variables displayed clear seasonal variations. Peaks occurred more frequently for all the pollutants during the cold period. NO_2_ and SO_2_ averages were higher during the cold period, while PM_10_ levels were similar during the two periods (See Appendix, Table A1). Tapp and wind speed were higher during the warm period. It rained more during the cold period. The pollutants never exceeded the daily nor annual South African Ambient Air Quality Standards (Figure A3, Appendix) [3,9]. However, PM_10_ and SO_2_ levels exceeded the more conservative daily World Health Organization (WHO) Air quality guidelines on 120 and 327 days, respectively (Figure A3) [3]. 

### 3.3. Excess Risk of Mortality

**Figure A5:**
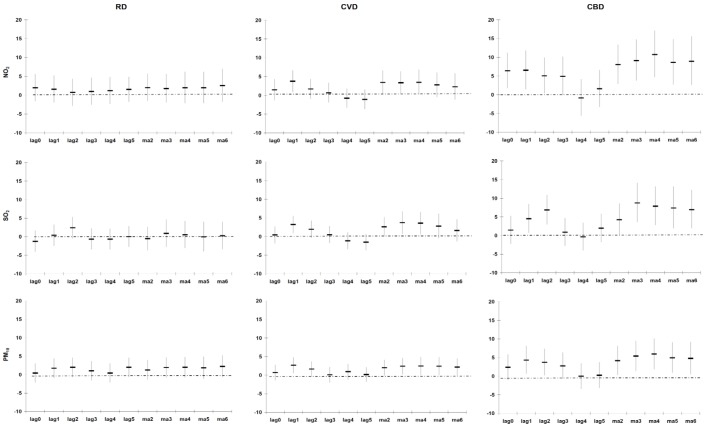
Percentage change (95% CI) in cause-specific mortality in Cape Town per inter-quartile range increase in PM_10_, NO_2_ and SO_2_ during 2001–2006 (entire year), adjusted for Tapp, rainfall, wind speed and public holidays.

**Figure A6:**
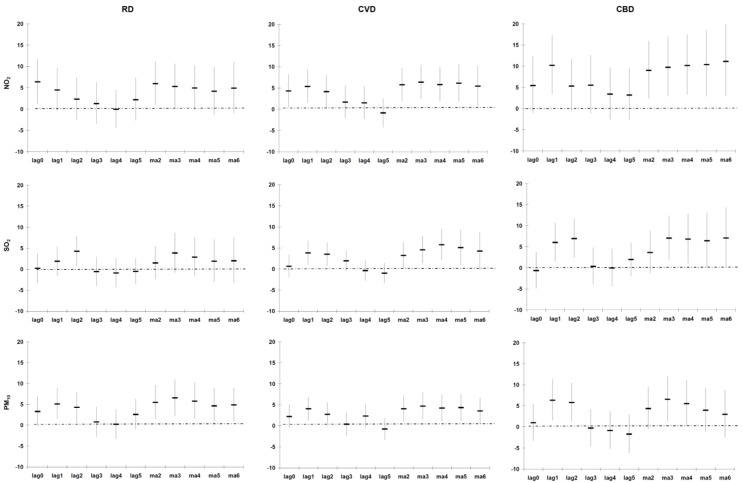
Percentage change (95% CI) in cause-specific mortality in Cape Town per inter-quartile range increase in PM_10_, NO_2_ and SO_2_ during 2001–2006 (warm period), adjusted for Tapp, rainfall, wind speed and public holidays.

**Figure A7:**
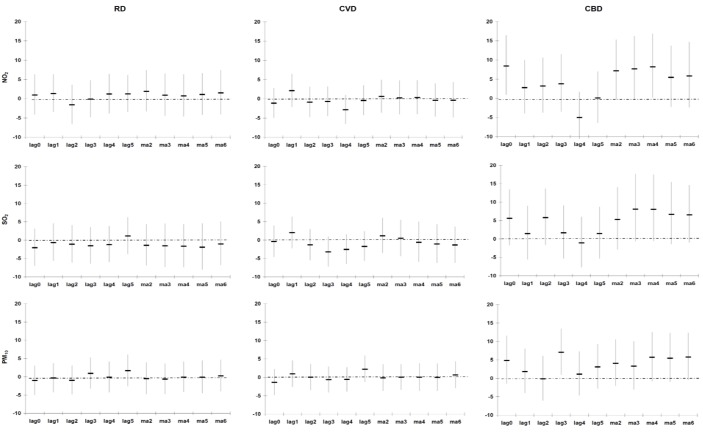
Percentage change (95% CI) in cause-specific mortality in Cape Town per inter-quartile range increase in PM_10_, NO_2_ and SO_2_ during 2001–2006 (cold period), adjusted for Tapp, rainfall, wind speed and public holidays.

Figure A5, Figure A6, Figure A7 (see Appendix) illustrate the % change in the cause-specific mortality per IQR increase in the different lags of PM_10_, NO_2_ and SO_2_ during 2001–2006 for the entire year, the warm and cold periods, after adjusting for Tapp, rainfall, wind speed and public holidays. In general the strongest significant association was observed between the CA2 of the pollutants (lowest AIC). CA2 of the pollutants was thus selected in the subsequent analyses.

For models that included data from the entire year, an IQR (12 μg/m^3^) increase in the CA2 of PM_10_ significantly increased CBD mortality in all areas by 4% (95%CI: 0.4 to 8%) (Table 2). No susceptible groups were identified for CBD mortality as the strength of associations was similar to the unstratified analyses. PM_10_ was significantly associated with CVD mortality in areas ≤10 km from any of the three monitoring sites, which was also stronger than for all areas. 

For the entire year, an IQR (12 μg/m^3^) increase in the CA2 of NO_2_ significantly increased CVD and CBD mortality in all areas by 3% (95% CI: 0.3 to 7%) and 8% (95% CI: 3 to 13%), respectively (Table 2). CBD mortality had a stronger association with the pollutants than with CVD mortality. The association between NO_2_ and CBD mortality was stronger for women than men, although the 95% CI was very wide. The strength of associations between NO_2_ and both outcomes was stronger in the ≤60 year group. Those living within 10 km from one of the three monitoring sites were more vulnerable to CVD mortality, whilst those living more than 10 km from the three monitoring sites were more vulnerable to CBD mortality.

**Table 2:** Association between air pollutant and cause-specific mortality in Cape Town, South Africa (entire year, 2001–2006).

		Respiratory Disease ^a^		Cardiovascular Disease ^a^		Cerebrovascular Disease ^a^	
Pollutant	Group	*N * ^b^	% ^c^ (95% CI)	*N * ^b^	% ^c^ (95% CI)	*N * ^b^	% ^c^ (95% CI)
**PM_10_**	**All**	13,407	1.3 (−1.4, 4.0)	21,510	2.0 (−0.1, 4.2)	7,573	4.1 (0.4, 8.1) *
	**Sex**						
	Women	6,030	0.9 (−3.0, 5.0)	11,145	2.3 (−1.7, 6.5)	4,212	2.6 (−4.4, 10.2)
	Men	7,377	1.5 (−2.0, 5.2)	10,364	2.0 (−0.6, 4.8)	3,359	4.7 (0.3, 9.4) *
	**Age**						
	≤60 years	6,128	3.4 (−0.5, 7.5)	5,986	2.3 (−0.7, 5.3)	2,090	5.3 (0.2, 10.6) *
	>60 years	7,279	−0.5 (−4.0, 3.1)	15,524	1.9 (−1.4, 5.3)	5,483	2.7 (−2.9, 8.6)
	**Distance**						
	≤10 km	6,964	3.2 (−0.5, 7.0)	12,894	4.3 (1.6, 7.2) *	4,569	3.6 (−1.2, 8.7)
	>10 km	6,443	−0.9 (−4.6, 3.0)	8,616	−1.4 (−4.6, 2.0)	3,004	4.9 (−1.1, 11.3)
**NO_2_**	**All**	13,398	2.0 (−1.6, 5.7)	21,488	3.4 (0.3, 6.6) *	7,565	8.0 (2.9, 13.4) *
	**Sex**						
	Women	6,026	3.0 (−2.4, 8.7)	11,136	1.6 (−4.1, 7.7)	4,208	10.2 (0.5, 20.8) *
	Men	7,372	1.2 (−3.9, 6.6)	10,351	3.8 (0.4, 7.2) *	3,355	7.3 (1.3, 13.5) *
	**Age**						
	≤60 years	6,123	4.1 (−1.2, 9.8)	5,981	4.8 (0.8, 8.9) *	2,089	9.4 (2.5, 16.6) *
	>60 years	7,275	0.2 (−4.5, 5.2)	15,507	1.5 (−2.9, 6.1)	5,476	6.3 (−1.2, 14.4)
	**Distance**						
	≤10 km	6,959	0.8 (−4.0, 5.9)	12,880	7.7 (3.6, 11.9) *	4,564	6.2 (−0.2, 13.1)
	>10 km	6,439	3.6 (−2.1, 9.6)	8,608	−2.8 (−7.1, 1.7)	3,001	10.7 (2.6, 19.4) *
**SO_2_**	**All**	13,393	−0.5 (−3.6, 2.6)	21,492	2.6 (0.1, 5.2) *	7,564	4.2 (0.0, 8.6)
	**Sex**						
	Women	6,025	−1.8 (−6.3, 2.9)	11,140	2.0 (−2.8, 6.9)	4,205	3.3 (−4.6, 12.0)
	Men	7,368	0.5 (−3.7, 4.9)	10,351	2.8 (−0.1, 5.9)	3,357	4.5 (−0.4, 9.7)
	**Age**						
	≤60 years	6,120	0.0 (−4.5, 4.8)	5,979	1.0 (−2.5, 4.6)	2,089	6.5 (0.9, 12.6) *
	>60 years	7,273	−1.2 (−5.8, 3.7)	15,513	4.3 (0.6, 8.2) *	5,475	1.3 (−4.9, 7.8)
	**Distance**						
	≤10 km	6,956	−0.9 (−5.2, 3.5)	12,884	5.3 (2.0, 8.8) *	4,565	4.2 (−1.2, 9.9)
	>10 km	6,437	−0.2 (−4.6, 4.5)	8,608	−1.4 (−5.3, 2.6)	2,999	4.2 (−2.3, 11.2)

^a^ Adjusted for public holidays, 2-day cumulative averages of 24-h apparent temperature, wind speed and rainfall. ^b^ Number of deaths included in the models, which is less than 13,439 RD; 21,569 CVD and 7,594 CBD deaths due to missing exposure data. ^c^ Percentage increase in risk per interquartile increase in the 2-day cumulative average: 12 µg/m^3^ PM_10_, 12 µg/m^3^ NO_2_, 8 µg/m^3^ SO_2_. * *p* < 0.05

**Table 3:** Association between air pollutant and cause-specific mortality in Cape Town, South Africa (warm period ^a^, 2001–2006).

		Respiratory disease ^b^		Cardiovascular disease ^b^		Cerebrovascular disease ^b^	
Pollutant	Group	*N * ^c^	% ^d^ (95% CI)	*N * ^c^	% ^d^ (95% CI)	*N * ^c^	% ^d^ (95% CI)
**PM_10_**	**All**	7,615	5.5 (1.4, 9.6) *	13,170	4.1 (1.0, 7.2) *	4,559	4.4 (−0.5, 9.5)
	**Sex**						
	Women	3,391	4.6 (−1.2, 10.8)	6,794	4.9 (−0.3, 10.4)	2,531	−1.5 (−10.2, 8.0)
	Men	4,224	5.6 (0.6, 10.8) *	6,376	3.5 (0.0, 7.3)	2,026	6.7 (0.9, 12.8) *
	**Age**						
	≤60 years	3,553	8.3 (2.8, 14.1) *	3,765	2.6 (−1.3, 6.6)	1,277	5.4 (−1.1, 12.4)
	>60 years	4,062	2.4 (−2.9, 79)	9,405	5.4 (1.0, 10.0) *	3,282	3.3 (−4.5, 11.7)
	**Distance**						
	≤10 km	3,925	7.2 (1.6, 13.0) *	7,843	6.1 (2.0, 10.3) *	2,731	6.0 (−0.4, 12.7)
	>10 km	3,690	3.6 (−2.1, 9.6)	5,327	1.2 (−3.1, 5.6)	1,828	2.1 (−5.3, 10.1)
**NO_2_**	**All**	7,606	6.0 (1.0, 11.2) *	13,148	5.8 (1.9, 9.8) *	4,551	9.0 (2.4, 16.0) *
	**Sex**						
	Women	3,387	6.8 (−0.5, 14.7)	6,785	3.9 (−3.0, 11.3)	2,527	5.8 (−5.9, 19.0)
	Men	4,219	5.1 (−1.6, 12.1)	6,363	6.6 (2.0, 11.3) *	2,022	10.3 (2.5, 18.8) *
	**Age**						
	≤60 years	3,548	10.7 (3.1, 18.9) *	3,760	7.0 (1.7, 12.6) *	1,276	9.4 (0.6, 18.8) *
	>60 years	4,058	1.9 (−4.6, 8.8)	9,388	4.4 (−1.0, 10.1)	3,275	8.6 (−1.2, 19.4)
	**Distance**						
	≤10 km	3,920	4.6 (−2.1, 11.9)	7,829	8.8 (3.8, 14.1) *	2,726	9.4 (0.9, 18.6) *
	>10 km	3,686	7.3 (0.2, 15.1) *	5,319	1.3 (−4.5, 7.4)	1,825	8.5 (−1.7, 19.7)
**SO_2_**	**All**	7,601	1.5 (−2.4, 5.5)	13,152	3.2 (0.1, 6.4) *	4,550	3.6 (−1.2, 8.7)
	**Sex**						
	Women	3,386	1.4 (−5.1, 8.3)	6,789	1.8 (−3.9, 7.8)	2,524	−1.9 (−10.8, 7.8)
	Men	4,215	1.7 (−3.6, 7.2)	6,363	3.8 (0.2, 7.7) *	2,024	5.7 (0.0, 11.8)
	**Age**						
	≤60 years	3,545	5.0 (−0.8, 11.1)	3,758	1.0 (−3.2, 5.4)	1,276	5.8 (−0.7, 12.8)
	>60 years	4,056	−1.8 (−7.6, 4.4)	9,394	5.7 (1.1, 10.5) *	3,274	0.9 (−6.3, 8.6)
	**Distance**						
	≤10 km	3,917	0.6 (−4.7, 6.3)	7,833	4.5 (0.4, 8.7) *	2,727	5.0 (−1.4, 11.8)
	>10 km	3,684	2.4 (−3.1, 8.3)	5,319	1.5 (−3.3, 6.4)	1,823	1.8 (−5.6, 9.7)

^a^ Warm period: January–April and September–December. ^b^ Adjusted for public holidays, 2-day cumulative averages of 24-h apparent temperature, wind speed and rainfall. ^c^ Number of deaths included in the models. ^d^ Percentage increase in risk per interquartile increase in the 2-day cumulative average: 12 µg/m^3^ PM_10_, 10 µg/m^3^ NO_2_, 7 µg/m^3^ SO_2. _ * *p* < 0.05.

**Table 4:** Association between air pollutant and cause-specific mortality in Cape Town, South Africa (cold period ^a^, 2001–2006).

		Respiratory disease ^b^		Cardiovascular disease ^b^		Cerebrovascular disease ^b^	
Pollutant	Group	*N * ^c^	% ^d^ (95% CI)	*N * ^c^	% ^d^ (95% CI)	*N * ^c^	% ^d^ (95% CI)
**PM_10_**	**All**	5,792	−0.5 (−4.8, 3.9)	8,340	−0.1 (−3.7, 3.6)	3,014	4.1 (−2.0, 10.6)
	**Sex**						
	Women	2,639	−1.8 (−8.0, 4.9)	4,351	−2.0 (−8.8, 5.3)	1,681	7.4 (−5.2, 21.6)
	Men	3,153	0.5 (−5.2, 6.6)	3,988	0.5 (−3.6, 4.8)	1,333	3.0 (−4.0, 10.6)
	**Age**						
	≤60 years	2,575	1.6 (−4.7, 8.4)	2,221	1.4 (−3.6, 6.6)	813	4.6 (−3.5, 13.3)
	>60 years	3,217	−2.2 (−7.9, 3.8)	6,119	−1.8 (−6.8, 3.5)	2,201	3.2 (−5.9, 13.1)
	**Distance**						
	≤10 km	3,039	2.1 (−3.9, 8.4)	5,051	2.4 (−2.3, 7.3)	1,838	1.0 (−6.5, 9.2)
	>10 km	2,753	−3.4 (−9.4, 3.0)	3,289	−4.0 (−9.4, 1.7)	1,176	8.8 (−1.1, 19.8)
**NO_2_**	**All**	5,792	1.9 (−3.3, 7.4)	8,340	0.6 (−3.6, 4.9)	3,014	7.2 (−0.4, 15.3)
	**Sex**						
	Women	2,639	1.4 (−6.3, 9.7)	4,351	−2.1 (−10.4, 7.0)	1,681	14.1 (−1.9, 32.6)
	Men	3,153	2.4 (−4.5, 9.8)	3,988	1.5 (−3.4, 6.6)	1,333	5.1 (−3.5, 14.6)
	**Age**						
	≤60 years	2,575	3.2 (−4.5, 11.4)	2,221	1.9 (−3.9, 8.0)	813	8.5 (−1.6, 19.5)
	>60 years	3,217	0.9 (−6.0, 8.4)	6,119	−0.8 (−6.8, 5.5)	2,201	5.3 (−5.8, 17.7)
	**Distance**						
	≤10 km	3,039	0.6 (−6.4, 8.0)	5,051	4.4 (−1.0, 10.2)	1,838	3.4 (−6.0, 13.7)
	>10 km	2,753	3.5 (−4.2, 11.8)	3,289	−5.4 (−11.7, 1.3)	1,176	13.7 (0.5, 28.7) *
**SO_2_**	**All**	5,792	−1.4 (−6.9, 4.4)	8,340	1.2 (−3.5, 6.0)	3,014	5.3 (−2.9, 14.1)
	**Sex**						
	Women	2,639	−4.8 (−12.6, 3.7)	4,351	1.5 (−7.4, 11.1)	1,681	12.6 (−3.8, 31.9)
	Men	3,153	1.5 (−6.1, 9.6)	3,988	1.0 (−4.4, 6.7)	1,333	2.7 (−6.4, 12.8)
	**Age**						
	≤60 years	2,575	−5.1 (−12.9, 3.5)	2,221	−0.2 (−6.5, 6.6)	813	8.3 (−3.7, 21.9)
	>60 years	3,217	1.6 (−5.9, 9.7)	6,119	2.5 (−4.1, 9.7)	2,201	2.1 (−9.5, 15.3)
	**Distance**						
	≤10 km	3,039	−1.0 (−8.5, 7.1)	5,051	5.4 (−0.8, 11.8)	1,838	2.4 (−7.7, 13.5)
	>10 km	2,753	−1.9 (−9.7, 6.6)	3,289	−5.2 (−12.1, 2.2)	1,176	9.8 (−3.4, 24.8)

^a^ Cold period: May–August. ^b^ Adjusted for public holidays, 2-day cumulative averages of 24-h apparent temperature, wind speed and rainfall. ^c^ Number of deaths included in the models. ^d^ Percentage increase in risk per interquartile increase in the 2-day cumulative average: 14 µg/m^3^ PM_10_, 13 µg/m^3^ NO_2_, 10 µg/m^3^ SO_2_. * *p* < 0.05.

For the entire year, an IQR (8 μg/m^3^) increase in the CA2 of SO_2_ significantly increased CVD mortality in all areas by 3% (95% CI: 0.1 to 5%) (Table 2). Similar vulnerable groups were identified for SO_2_ than for PM_10_ and NO_2_.

The effect estimate of the subgroup analyses had wider 95% CI due to smaller sample sizes. NO_2_ displayed stronger associations with CVD and CBD mortality than SO_2_ and PM_10_. CBD mortality had the strongest association with the pollutants, compared to CVD and RD mortality. 

The associations were stronger in the warm period than the entire year (Table 3). In the warm period, PM_10_ had a significant association with RD and CVD mortality in all areas, with the former slightly stronger. The strength of associations between PM_10_ and both outcomes was stronger for those living within 10 km from the monitoring sites. For RD mortality, the ≤60 year group was more vulnerable to increases in PM_10_, whilst this was the case for the >60 year group and CVD mortality. PM_10_ was significantly associated with CBD mortality amongst men. In the warm period, NO_2_ had significant associations with CBD, RD and CVD mortality (in order of association strength). For all three outcomes, the younger age group was more vulnerable to NO_2_ increases. For CVD and CBD mortality, men and those living within 10 km from any of the three monitoring sites were more vulnerable to NO_2_ increases. Those living more than 10 km away from the monitoring sites were more at risk of RD mortality. In the warm period, SO_2_ was significantly associated with CVD mortality. As with PM_10_, the strength of associations between SO_2_ was stronger for those living within 10 km from the monitoring sites and in the older age group. Men were also more vulnerable to CVD mortality. NO_2_ displayed stronger associations than PM_10_ and SO_2_.

None of the pollutants were associated with any of the three outcomes in the cold period (Table 4). The associations were weaker for RD and CVD mortality in all areas compared to those of the entire year and the warm period. Similar results were observed for CBD mortality in the entire year and the warm and cold periods. The observed associations in the case-crossover analyses were confirmed in the GAM analyses (see Appendix, Table A2). 

## 4. Discussion and Conclusions

Daily PM_10_, NO_2_ and SO_2_ levels in Cape Town City Centre, Goodwood and Tableview are comparable or even lower than to those in North America and Western Europe, and much lower than levels observed in South America, Asia and North Africa [3].

Our estimate of association (excess mortality risk) between PM_10_, and RD, CVD and CBD mortality of 1.1% (−1.1%; 3.3%), 1.7% (−0.1%; 3.5%) and 3.2% (0.3%; 6.2%) following a 10 µg/m^3^ increase (entire year data), respectively, is higher than those observed in a meta-analysis conducted on studies from North America, South America, Europe, the Eastern Mediterranean, South East Asia and the Western Pacific [2]. The meta-analysis reported excess mortality risks of 1.0% (0.5%; 1.4%), 0.6% (0.2%; 1.1%) and 0.4% (0.0%; 0.8%) for RD, CVD and CBD mortality following a 10 µg/m^3^ increase in PM_10_, respectively. However, heterogeneity in the effect estimates was observed in the different individual and multiple city studies. 

Our estimate of association between NO_2_ and RD mortality of 1.7% (−1.3%; 4.7%), following a 10 µg/m^3^ increase (entire year data) is the same as that of the meta-analysis 1.7% (1.3%; 2.1%) [2]. Our estimate of association between NO_2_, and CVD and CBD mortality of 2.6% (0.2%; 5.0%) and 6.6% (2.4%; 11.0%) following a 10 µg/m^3^ increase (entire year data), respectively, is higher than those observed in the meta-analysis: 0.7% (0.5%; 0.8%) and 0.8% (0.2%; 1.4%), respectively [2]. As for PM_10_, heterogeneity in the effect estimates was observed in the different individual and multiple city studies. 

Our estimate of association between SO_2_, and RD, CVD and CBD mortality of −0.7% (−4.5%; 3.3%), 3.3% (0.1%; 6.5%) and 5.3% (0.0%; 10.9%) following a 10 µg/m^3^ increase (entire year data), respectively, is in general higher than those observed in the meta-analysis [2]. The meta-analyses reported excess mortality risks of 0.3% (0.0%; 0.6%), 2.0% (0.6%; 3.5%) and 2.1% (0.7%; 3.6%) for RD, CVD and CBD mortality following a 10 µg/m^3^ increase in SO_2_, respectively [2]. As for NO_2_ and PM_10_, heterogeneity in the effect estimates was observed in the different individual and multiple city studies. 

Two multicity studies in the USA investigated whether warm and cold seasonal differences exist in the association between PM_10_ and natural all-cause mortality and one multicity study investigated this for SO_2_, and RD and CVD mortality in Europe [2]. No seasonal differences were observed between PM_10_ and all-cause natural mortality, but slightly stronger SO_2_ effects on RD and CVD mortality in the warmer months. A study by Goldberg *et al.* found higher PM_2.5_, NO_2_ and SO_2_ effects on natural all-cause mortality amongst ³65 year olds with diabetes in the warm season in Montreal, Canada, compared to the entire year [16]. A study in Belgium also reported stronger association between PM_10_ and natural all-cause mortality during spring and summer, compared to autumn and winter [17]. Another study observed significant increases in PM_10_ effect estimates on natural all-cause mortality in Bangkok, Thailand and Wuham, China in the warm season, but decreases in Hong Kong, China and to a lesser extent in Wuham, China [18]. Seasonal differences in air pollution effect estimates may have substantial implications for the setting of air pollution health standards.

Speculations about the mechanisms underlying the much stronger association between mortality and air pollution exposure during warmer periods, even though the levels reach may be higher in winter, may be due to the difference in the component-specific toxicity of air pollution across the temperature range. A study that exposed isolated macrophages of rats to ambient PM_10_ collected during winter, spring and summer (in Amsterdam, Lodz, Oslo and Rome) showed that samples collected in summer were more potent at inducing inflammatory cytokines (interleukin 6 and tumor necrosis factor a) [19]. The higher effects during the warmer months may also be due to more time spent outdoors or because of a closer similarity between indoor and outdoor air pollution. Lower background mortality in the warmer months, thus resulting in a larger pool of susceptible people, may also explain the higher relative effects observed in these months [20]. However, currently it is not clear whether air pollutants are effect modifiers and/or confounders of the temperature-mortality relationship [13].

Anderson *et al.* postulated reasons for the heterogeneity in air pollutant effects estimates in different studies, which include variations in exposure measurement error, toxicity of the pollution, different ages and vulnerability of the population, differences in the way the exposure and outcome series had been assembled, statistical approaches selected, specification of meteorological variables, approaches to control time-varying confounders and publication bias [2]. Anderson *et al.* also acknowledged that the relative contributions of these factors were (and still are) poorly understood [2]. 

Issues affecting the vulnerability of the population in Cape Town are numerous. South Africa has the highest *per capita* annual risk of TB disease of comparably sized countries globally, and its communities have extremely high TB transmission rates. The rates of TB infection of children and adolescents are now similar to those reported 100 years ago in Europe long before chemotherapy became available [21]. This means many people already have compromised respiratory systems. Use of polluting indoor fuel has been linked to increase in the incidence of respiratory infections, including pneumonia, tuberculosis and COPD, low birth weight, cataracts, cardiovascular events and all-cause mortality both in adults and children, which adds to the vulnerability of the population [22].

Advantages of our study include accurate air pollution and meteorological data. Our study results from the case-crossover models were confirmed in time series models and our lag structure with main effects on cause-specific mortality occurring within 2 days is compatible with other studies [2].

A review of the quality of cause of death data from 115 WHO member countries, published in 2005, categorized South Africa as having low quality cause of death data based on a combination of a more than 20% of deaths being ill-defined deaths and less than 70% of deaths being registered [23]. The completeness of death registration for adults in Cape Town during the period 2001–2006 was estimated to be 96%, with the exception of 2005 where the completeness was 84%, about 55% for children 0–4 years, and about 70% for infants [8]. A report by Bradshaw *et al.* concluded that South Africa now fits into the category of countries with medium rather than poor quality [24]. Many European countries are in the medium quality category. Thus health outcome misclassification is possible, but it is unlikely to be related to air pollution. Our confidence in the accuracy of the mortality data is supported by the clear seasonal trends.

A limitation of all case-crossover and time series epidemiological studies is the assumption that the ambient air pollution and meteorological variables measured at a few sites are the same across the entire city, which might have resulted in a measurement error. This exposure misclassification is non-differential and should bias the effect estimates towards the null [25]. In general we observed stronger associations with those living within 10 km from the three monitoring sites.

Another limitation is that the meteorological data were collected at the airport, which is 25 km from the city center. This may influence how well the meteorological variables are correlated with the pollutants, and eventually affect the robustness of the study results.

Other limitations are that ozone data were not sufficiently available from the Goodwood site (≥70% of 1-hour values missing in 2001–2006) and that the chemical composition of PM_10_ was not available. Ozone is a potentially important confounder, especially in the warm period when levels are higher [2].

Much has been learned from epidemiological studies regarding the adverse health effects of outdoor air pollution in the past 25 years. Nonetheless, important research gaps remain that are critical to public policy, such as identifying the most toxic constituents of the pollutant mix and sources contributing to it, pinpointing social conditions, genetic factors and pre-existing diseases that increase vulnerability, teasing out the how much long-term exposure, especially during childhood, affect the development of chronic, life-threatening disease [26]. The answers to these questions may well be different in Africa than in the developed world, where to date the vast majority of epidemiological studies have been conducted. The current uncertainty regarding the shape and the slope of the air pollution concentration–response function for long-term exposure and RD, CVD and CBD mortality is also important for Africa, where levels may exceed those observed in the USA and Europe.

Norman *et al.* estimated that 3.7% of CVD mortality in South Africa is attributed to outdoor PM_2.5_ [27]. The PM_2.5_ response function of the American Cancer Society (ACS) cohort study was applied in this estimation [28]. Long-term exposure in cohort studies indicate stronger associations with health outcomes than short-term case-crossover or time series epidemiological studies [3]. Cohort studies in South Africa might thus also indicate stronger effects than that of the ACS study. This will mean that the attributable fraction due to outdoor air pollution may be much higher in South Africa than estimated by Norman *et al.* [27].

In conclusion, this study is very relevant in stressing the importance of improving air quality in South Africa. The RD, CVD and CBD mortality risks associated with ambient air pollution exposure is in general much higher in Cape Town than those reported in studies from the USA and Europe. Whether our results are due to statistical chance or consistent over time can only be revealed by continuation of monitoring of the associations over time in the future.

## Figures and Tables

**Figure A8:**
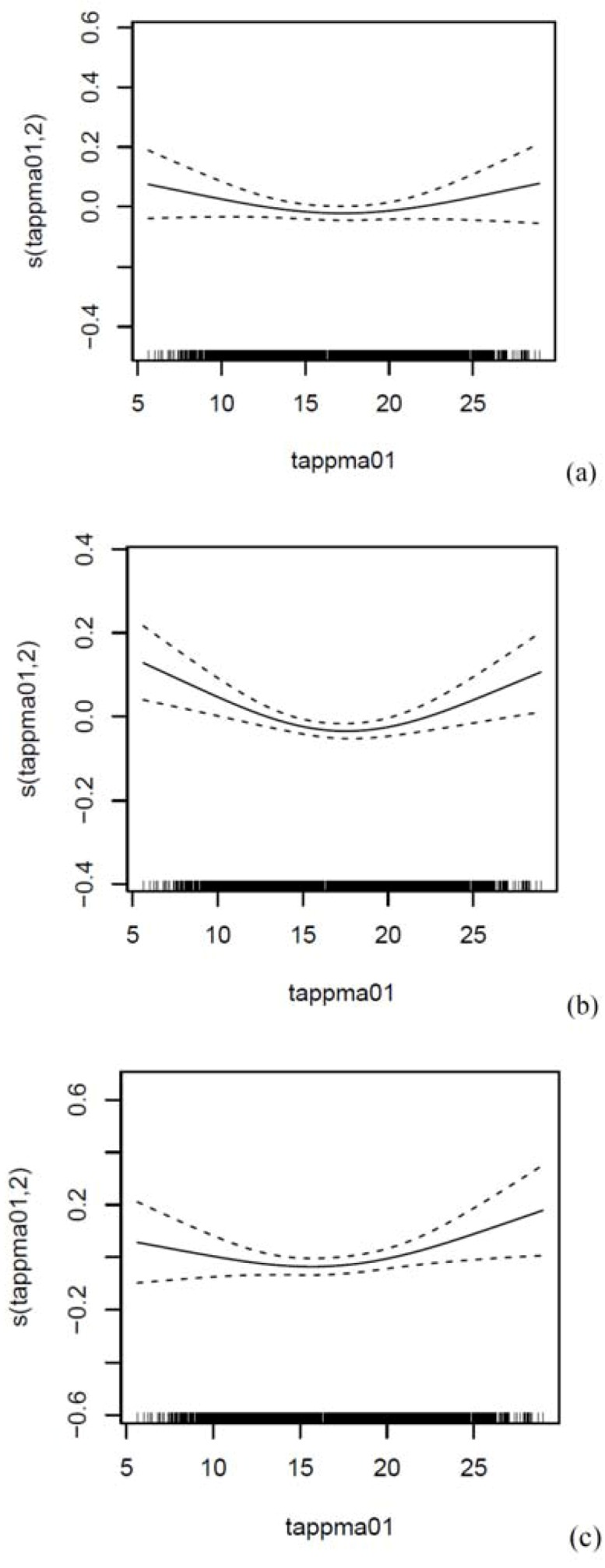
Smoothed relationship between the 2-day cumulative average of Tapp (entire year) and (**a**) RD, (**b**) CVD and (**c**) CBD mortality in Cape Town, South Africa during 2001–2006. Adjusted for the 2-day cumulative average of PM_10_, NO_2_ or SO_2_, and long term seasonal trends, public holidays, day of the week, 2-day cumulative averages of 24-h wind and rain. K = 3 for Tapp, K = 30 for time, *i.e.* 5df/year.

**Figure A9:**
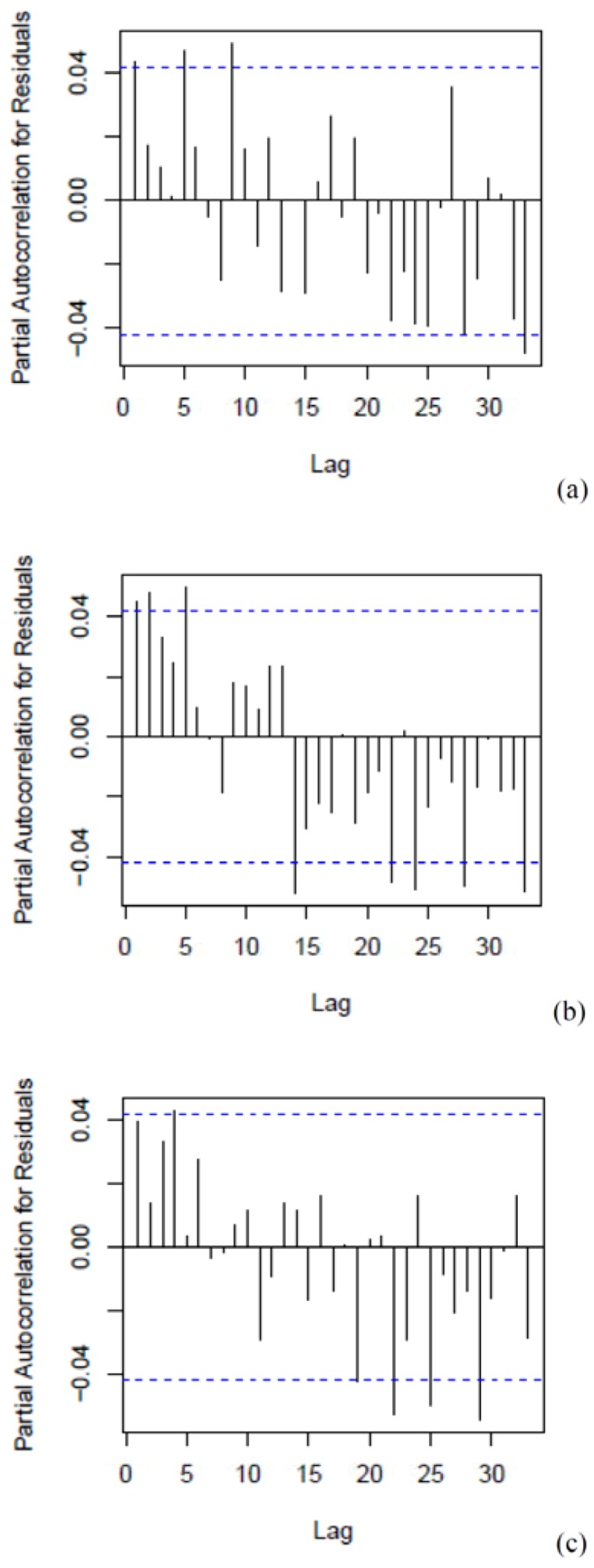
Partial autocorrelation function residuals for the generalised additive Poisson time-series regression models (entire year): 2-day cumulative average of PM_10_ and RD (**a**), CVD (**b**) and CBD (**c**) mortality in Cape Town, South Africa during 2001–2006. Adjusted for the 2-day cumulative average of PM_10_, NO_2_ or SO_2_, and long term seasonal trends, public holidays, day of the week, 2-day cumulative averages of 24-h apparent temperature, wind and rain. K = 30 for time, *i.e.* 5df/year.

**Figure A10:**
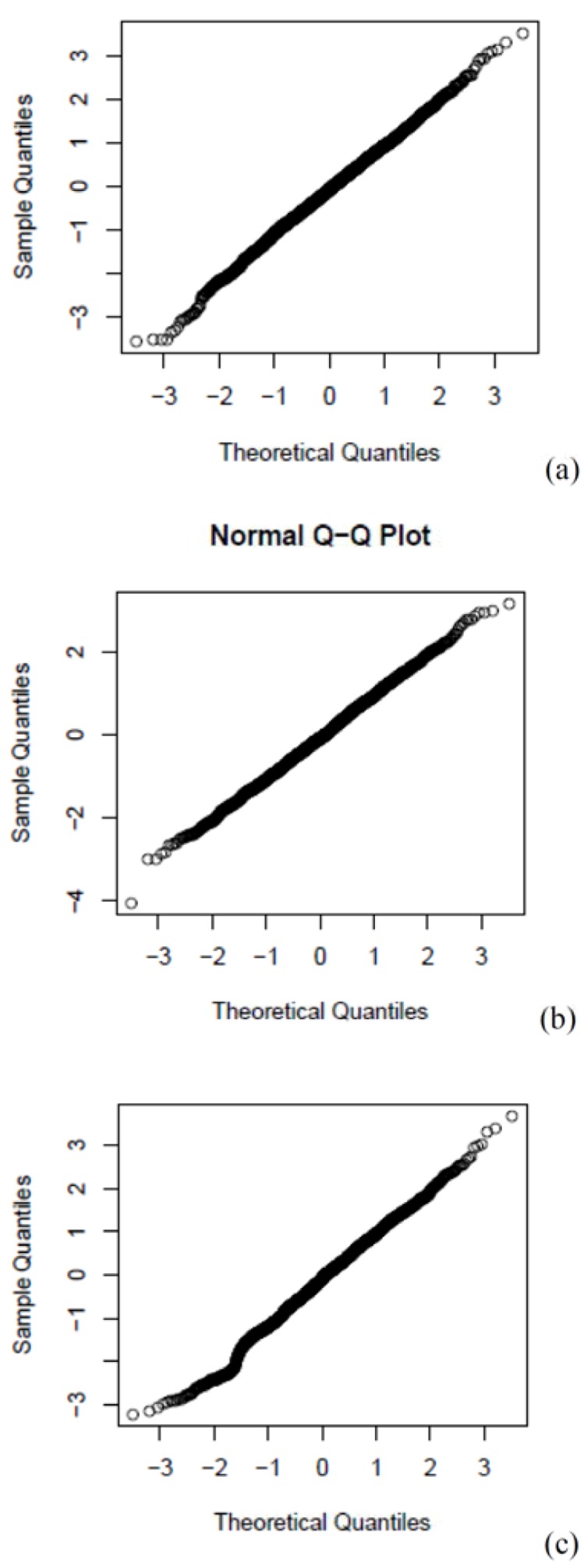
Normal Q-Q plot for the generalised additive Poisson time-series regression models (entire year): RD (**a**), CVD (**b**) and CBD (**c**) mortality in Cape Town, South Africa during 2001–2006. Adjusted for the 2-day cumulative average of PM_10_, NO_2_ or SO_2_, and long term seasonal trends, public holidays, day of the week, 2-day cumulative averages of 24-h apparent temperature, wind and rain. K = 30 for time, *i.e.* 5df/year.

## Appendix

### 1. Study Location

Cape Town is the second most populous city in South Africa, forming part of the City of Cape Town metropolitan municipality. The city has an estimated population of 3.5 million [1]. The Cape Peninsula has a subtropical Mediterranean climate. In winter cold fronts come from the Atlantic Ocean with heavy precipitation and strong north-westerly winds. Summer months are warm and dry with frequent strong winds from the south-east (Indian Ocean) and the north (semi-arid Karoo interior).

### 2. Mortality Data

Although cause of death data had been collected in Cape Town for more than 100 years, the system has been revamped in recent years to provide the city with more relevant information. Cape Town has an ongoing system of collecting cause of death statistics based on copies of death notifications collected from the local offices of the Department of Home Affairs and supplemented by information collected from the local mortuaries [2]. The underlying cause of death is identified and coded using a shortlist based on the *International Classification of Diseases, 10th Revision*. The cause of death coding is done by trained clerks at Cape Town who also capture the data into a customised data base.

### 3. Air Pollution Data

Monitoring in Cape Town commenced in 1958 with the introduction of the first monitoring stationing measuring SO_2_ and smoke [3]. Over the years the data have been extensively used and reported on by researchers in various surveys and studies [3]. A sophisticated monitoring network was established in Cape Town from the mid 1980s. During 2001-2006 Cape Town had nine fixed and two mobile air quality monitoring stations spread across the metropole. During 2007–2012 three more stations were added. Figure A1 illustrates the location of the fourteen air quality monitoring stations in 2012.

The National Environmental Management: Air Quality Act of 2004 requires the monitoring of PM_10_, NO_2_ and SO_2_ [5]. These three air pollutants were included in the study and measured at the City Centre, Goodwood and Tableview sites. Data from the other sites were not used due to the large number of days with missing values.

The two sites at the City Hall and the Drill Hall (during 2001-2006) were located close to each other and were grouped together as the City Centre site during this investigation. The City Centre site is located in an urban area next to busy roads. Tableview is located in a residential area, but still relatively close to a busy highway and an oil refinery. Goodwood is also located in a residential area, but further from the highways and closer to a light industrial area.

O_3_ was measured at Molteno and Goodwood and CO was measured at the City Centre and Goodwood. These two pollutants were not included in this study due to the large number of days with missing values (≥70% of 1-hour values missing in 2001–2006).

### 4. Air Pollution Sources in Cape Town

Exposure to outdoor air pollutants is essentially beyond the control of individuals and requires action by public authorities at the national, regional and even international levels. Outdoor air quality remains a key issue in Cape Town [6]. The main sources of PM_10_ are diesel vehicles, wood and coal burning for household heating and cooking fuel, dust from construction activities and unpaved roads, as well as wind-blown dust. The main sources of NO_2_ and SO_2_ are diesel vehicles and coal burning for household fuel, as there are few large industries in the city [6].

### 5. Apparent Temperature

Apparent temperature is a construct intended to reflect the physiological experience of combined exposure to humidity and temperature and thereby better capture the response on health than temperature alone [7].

Saturation vapour pressure

= 6.112 × 10^(7.5 × temperature °C/(237.7 + temperature °C)^ (1)

Actual vapour pressure

= (relative humidity (%) × saturation vapour pressure)/100
(2)

Dew point temperature °C

= (−430.22 + 237.7 × ln(actual vapour pressure))/(−ln(actual vapour pressure) + 19.08)
(3)

Apparent temperature °C

= −2.653 + (0.994 × temperature °C) + 0.0153 × (dew point temperature °C)
(4)

### 6. Generalised Additive Poisson Time-Series Regression Models

The linearity of the association between Tapp and a cause-specific outcome was confirmed in generalised additive Poisson time-series regression models (GAM) with the use of the *gam* procedure, *mgcv* package in R statistical software (R Development Core Team, 2010). Smoothing splines of calendar time with 2 to 8 degrees of freedom per year (df/year) were used to control for long-term trend and seasonality, with between 4 and 8 df/year the optimum for the entire year, and the warm and cold periods. Models were run with linear and non-linear terms of Tapp, the latter as a smoothing spline function with 3, 5 and 7 df. We investigated whether the non-linear term of Tapp improved the models by conducting log-likelihood ratio tests. We decided not to use a spline for Tapp, as the splines were insignificant, did not add value to the models and the pollutant model estimates were not influenced, whether 3, 5 or 7 df (Figure A8). The GAM models were also adjusted for day of the week (dummy variable), public holidays (binary variable), wind speed and rainfall (linear variables).

A spline function, defined by piecewise polynomials, has a flexible shape that is useful for adjusting for non-linear effects. The smoothness of a spline is a function of the number of degrees of freedom. Unmeasured, unknown and potentially variable seasonal and long term patterns need to be controlled for adequately in GAM models, whilst still leaving sufficient information from which to estimate air pollutant effects. Randomness is one of the key assumptions in determining whether a univariate statistical process is in control. This randomness is ascertained by computing autocorrelations for data values at varying time lags. Our data were assumed to be random when such autocorrelations were near-zero for any and all time-lag separations. Long-term trends and seasonality are controlled adequately at the optimum df/year when the partial aurocorrelation residuals summed to zero (Figure A9). The normal quantile-quantile residual plots for checking the GAM model fitting process were very close to a straight line, suggesting reasonable distributional assumption (Figure A10).

The case-crossover design has some advantages over the Poisson time-series design [8]. In GAM analysis, the population at risk must be very large relative to the daily number of events and the composition and size of the population at risk must not co-vary with the exposure of interest. The later assumption may not be fully met whenever the susceptible portion of the total population at risk may be increased by the cumulative effects of prior exposures or decreased by the adverse effects of prior exposures (harvesting). The case-crossover design avoids both problems as the outcome is on an individual level and not a population level (daily number of events).

**Table A1:** Air pollutant levels and meteorological conditions measured at three sites in Cape Town, South Africa, 2001–2006.

	Area Levels			Aggregate Levels		
	City	Goodwood	Tableview	Entire Year	Warm Period ^a^	Cold Period ^a^
Number of days				2,191	1,453	738
PM_10_ (µg/m^3^)						
Number of days with missing data	25	18	31	0	0	0
Mean ± SD	22.9 ± 10.9	26.2 ± 11.4	30.5 ± 14.8	27.4 ± 12.1	26.6 ± 10.9	28.9 ± 14.0
Range	3.8-76.5	3.3-96.3	1.3-216.7	3.6-101.5	3.6-101.5	6.8-94.3
IQR	13	14	16	14	13	16
NO_2_ (µg/m^3^)						
Number of days with missing data	16	35	60	1	1	0
Mean ± SD	36.0 ± 15.4	16.5 ± 9.6	13.6 ± 7.4	25.1 ± 11.8	22.2 ± 9.7	30.7 ± 13.5
Range	3.4-116.4	1.8-71.3	1.1-66.1	5.9-88.8	5.9-73.0	7.1-88.8
IQR	19	10	9	13	11	14
SO_2_ (µg/m^3^)						
Number of days with missing data	21	267	34	1	1	0
Mean ± SD	14.1 ± 10.5	9.1 ± 6.0	10.8 ±10.9	12.6 ± 8.2	11.6 ± 7.5	14.7 ± 8.9
Range	0-143.6	0.8-46.7	0.2-83.3	0.9-59.3	0.9-59.3	1.3-47.4
IQR	11	6	8	9	7	11
Tapp (°C)						
Number of days with missing data	-	-	-	2	2	0
Mean ± SD	-	-	-	16.9 ± 5.1	19.2 ± 4.3	12.2 ± 2.9
Range	-	-	-	5.3-29.7	6.6-29.7	5.3-21.1
IQR	-	-	-	8	6	4
Rain (mm)						
Number of days with missing data	-	-	-	4	2	2
Mean ± SD	-	-	-	1.2 ± 3.9	0.7 ± 2.6	2.3 ± 5.5
Range	-	-	-	0-60.0	0-33.2	0-60.0
IQR	-	-	-	0.2	0	1.6
Wind speed (km/h)						
Number of days with missing data	-	-	-	1	1	0
Mean ± SD	-	-	-	17.3 ± 7.6	19.0 ± 7.2	13.7 ± 2.9
Range	-	-	-	2.2-46.4	4.0-46.4	2.2-38.5
IQR	-	-	-	2.7	9.7	8.6

^a^ Warm period: January-April and September-December, cold period May-August. IQR: Inter-quartile range. SD: Standard deviation

**Table A2:** Generalised additive Poisson time-series regression models for association between PM_10_, NO_2_ and SO_2_, and cause-specific mortality.

	Respiratory Disease ^a^					Cardiovascular Disease ^a^					Cerebrovascular Disease ^a^				
Period	n ^b^	IQR	% ^c^	(95% CI)		n^b^	IQR	%^c^	(95% CI)		n ^b^	IQR	% ^c^	(95% CI)	
**All year**															
PM_10_	2,181	12	0.7 (−1.8, 3.2)			2,181	12	1.7 (−0.2, 3.7)			2,181	13	4.1 (0.4, 8.0) *		
NO_2_	2,179	12	1.5 (−1.8, 5.0)			2,179	13	3.5 (0.7, 6.4) *			2,179	12	6.7 (1.8, 11.8) *		
SO_2_	2,179	8	−0.3 (−3.2, 2.6)			2,179	8	1.9 (−0.3, 4.2)			2,179	8	3.1 (−0.9, 7.2)		
**Warm ^d^**															
PM_10_	1,446	12	4.3 (0.7, 8.1) *			1,446	12	3.4 (0.7, 6.2) *			1,446	12	4.5 (−0.3, 9.5)		
NO_2_	1,444	10	5.9 (1.3, 10.6) *			1,444	10	4.8 (1.5, 8.3) *			1,444	10	7.6 (1.4, 14.3) *		
SO_2_	1,444	7	1.3 (−2.3, 5.0)			1,444	7	2.6 (−0.1, 5.4)			1,444	7	3.2 (−1.5, 8.1)		
**Cold ^e^**															
PM_10_	735	14	−1.5 (−5.7, 2.9)			735	14	−1.0 (−4.4, 2.6)			735	14	3.5 (−2.8, 10.2)		
NO_2_	735	13	1.7 (−3.4, 7.1)			735	13	0.8 (−3.2, 4.9)			735	13	6.9 (−0.8, 15.2)		
SO_2_	735	10	−0.1 (−5.4, 5.4)			735	10	0.6 (−3.7, 5.0)			735	10	4.3 (−3.6, 12.8)		

^a^ Adjusted for long term seasonal trends, public holidays, day of the week, 2-day cumulative averages of 24-h apparent temperature, wind and rain. ^b^ Number of days. ^c^ Percentage increase in risk and 95% confidence intervals per inter-quartile increase in the 2-day cumulative average of a pollutant (in µg/m^3^). ^d^ Warm period: January-April and September-December. ^e^ Cold period May-August. * *p* < 0.05.
